# The impact of Vitamin D Replacement on Glucose Metabolism

**DOI:** 10.12669/pjms.296.3891

**Published:** 2013

**Authors:** Parildar H, Cigerli O, Unal DA, Gulmez O, Demirag NG

**Affiliations:** 1 Parildar H, Department of Family Medicine, Baskent University, Turkey.; 2 Cigerli O, Department of Family Medicine, Baskent University, Turkey.; 3 Unal DA, Department of Endocrinology, Baskent University, Turkey.; 4 Gulmez O, Family Practitioner, Department of Family Medicine, Baskent University, Istanbul Hospital, Istanbul, Turkey.; 5 Demirag NG, Department of Endocrinology, Baskent University, Turkey.

**Keywords:** Vitamin D deficiency, Prediabetes, Insulin resistance

## Abstract

***Objective: ***We investigated the impact of vitamin D supplementation on glucose metabolism in Vitamin D-deficient patients with prediabetes.

***Methods:*** A total of 66 subjects with the mean ages 52.2±9.9 years were included in this prospective and a 6-month follow-up study between 2008-2010. Vitamin D deficient patients (<25ng/ml) were supplemented with oral Vitamin D.

***Results:*** Vitamin D deficiency (<25ng/ml) was found in 93.9% of the patients. Post replacement Vitamin D levels increased significantly and insulin, HbA1c, and HOMA-IR decreased significantly following Vitamin D replacement.

***Conclusion*** *:* We found Vitamin D deficiency was very common in our study population. In Vitamin D deficient patients, supplementation treatment improved insulin resistance and glycemic parameters. Vitamin D replacement may be a promising intervention for the primary prevention of insulin resistance syndromes.

## INTRODUCTION

Prediabetes has become one of the most important topics of preventive medicine in recent years. Long-term complications of diabetes may start to develop even in prediabetic period.^1^ Currently, the most important and basic intervention for preventing diabetes is lifestyle modifications including physical activity and dietary interventions.^2^

Vitamin D deficiency is one of the most researched topics in recent years.^3^^,^^4^ In cross-sectional and epidemiological studies, the relation of Vitamin D deficiency with DM type I, metabolic syndrome, obesity, cardiovascular diseases, hypertension and mortality associated possibly due to this vitamin role in insulin resistance, secretion and inflammatory process were reported.^5^^-^^11^ It has also been shown in various studies that deficiency in serum 25(OH)D levels decrease insulin secretion by reducing calcium absorption and therefore causing secondary hyperparathyroidism and increase peripheral insulin resistance.^12^ Therefore Vitamin D may be considered as a factor affecting the insulin resistance directly or indirectly. 

It is a fact that Vitamin D deficiency is common globally, including in sunny countries like our country.^13^^-^^15^ However, the number of studies looking at the effect of Vitamin D replacement on glycemic parameters are limited.

This study aimed to investigate the relation of vitamin D deficiency with metabolic parameters in patients with prediabetes and the effects of vitamin D on glucose metabolism, by replacement treatment among those with vitamin D deficiency.

## METHODS

A total of 66 prediabetic patients who applied to the outpatient clinics of Baskent University Istanbul Hospital between the years of 2008 and 2010 were included in our prospective, interventional study. Study subjects were classified as prediabetic by the American Diabetes Association (ADA) proposed diagnostic criteria for prediabetes (defined as IFG and IGT; fasting plasma glucose levels between 100-125 mg/dl and 140 to 199 mg/dl at second hour of 75 g glucose tolerance test, respectively. Insulin resistance was defined as the Homeostasis Model Assessment of Insulin Resistance (HOMA-IR) > 2.5. Patients using Vitamin D and/or calcium supplements, having diabetes, bone metabolism disorder and liver and kidney failure were excluded from the study. Informed consents were obtained from all patients. Approval of the University’s Ethical Committee was received (protocol no: KA08/129).

Serum 25(OH) D levels below 25 ng/ml were considered as deficiency, while above 25 ng/ml were considered as sufficiency^.^^16^ As there is still no consensus for the threshold of Vitamin D sufficiency regarding its extraskeletal effects and also these levels are not adjusted according to different areas in the world, we accepted the sufficiency threshold of Vitamin D levels as to maintain PTH levels below 45 pg/ml in our study. Vitamin D deficient individuals were supplemented with 2x300.000 IU Vitamin D3 (po) in one month as bolus followed by 1x800-1000 IU/d cholecalciferol plus elementary calcium of 1200 mg/d po as maintenance treatment. Medical nutrition therapy and a minimum of 150 minutes of aerobic exercises per week were recommended by dietician to all subjects and laboratory and anthropometric measurements repeated following 6 months. 

SPSS 16.0 (SPSS version 16.0, SPSS Inc. Chicago, IL, USA) package was used in statistical analyses. Mann Whitney U test was used for comparison of the parameters with nonparametric variables. Paired Sample T test or Wilcoxon test were used for analysis of intragroup comparisons. Spearman correlation analysis was performed to reveal the relationship of 25(OH)D levels with laboratory parameters. Data were expressed as mean and standard deviation or as number and percentage where appropriate. The results were evaluated at the level of significance p<0.05.

Serum 25(OH)D levels were measured with a chemiluminescent immunassay method (CMIA) (Architect i1000 system, Abbott, USA). Normal ranges were between (NA): 15.7-60.3 ng/ml (summer), 8.8-46.3 ng/ml (winter). Insulin levels were analysed with CMIA (Architect i1000 system, Abbott, USA), NA: 2.6-24.9 μU/ml. Serum PTH levels were measured with electrochemiluminescent immunassay method (ECLIA) (Architect i2000 system, Abbott, USA), NA:15-68pg/ml. HbA1c was measured by turbidimetric assay method (C4000, Architect cSystem, Abbott, USA). Plasma glucose levels were measured with enzymatic colorimetric assay (C8000 system, Abbott, USA).

## RESULTS

The mean ages of the patients were 52.2±9.9 years (range 31-76) and the percentage of female patients was 71.3% (n= 51). Table-I shows andropometric and basal metabolic parameters of study group.

**Table-I:** Baseline anthropometric measurements of the study group

*Study population (n=66)*	*(mean/SD)*	*range*
Age (year)	52.2±9.9	31-66
Height (cm)	163.5±9.0	150-191
Weight (kg)	80.9±16.0	50-130
BMI (kg/m²)	30.3±5.1	20.5-48.3
Waist circumference (cm)	98.9±12.0	69-127

**Table-II:** The analysis of baseline and post-replacement metabolic parameters in the study group (Paired Sample T test or Wilcoxon test were used for analysis of intragroup comparisons).

*Study population (n=66)*	*Initial values*	*Final values*	*p*
Vitamin D (ng/ml)	15.1±6.6	29.4±10.8	0.000
HbA1c (%)	5.7±0.5	5.5±0.4	0.027
Insulin (uI/ml)	15.0±7.0	12.5±5.7	0.037
HOMA-IR	4.1±1.9	3.3±1.5	0.031
PTH (pg/ml)	63.0±19.9	48.5±16.9	0.017
BMI (kg/m²)	28.6±4.9	27.9±4.4	0.149

**Fig.1:**
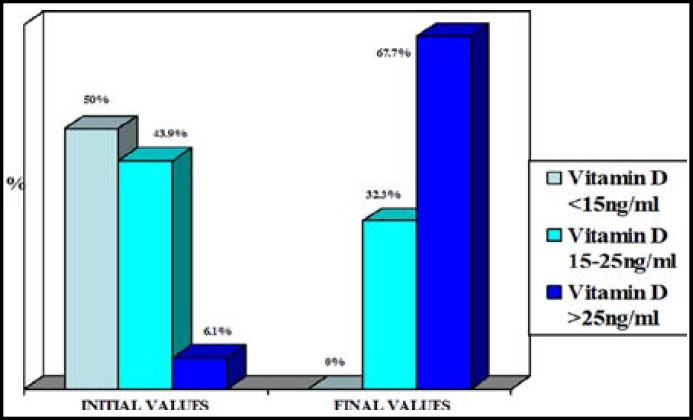
Distribution of baseline and post-replacement vitamin D levels (n=66).

**Fig.2:**
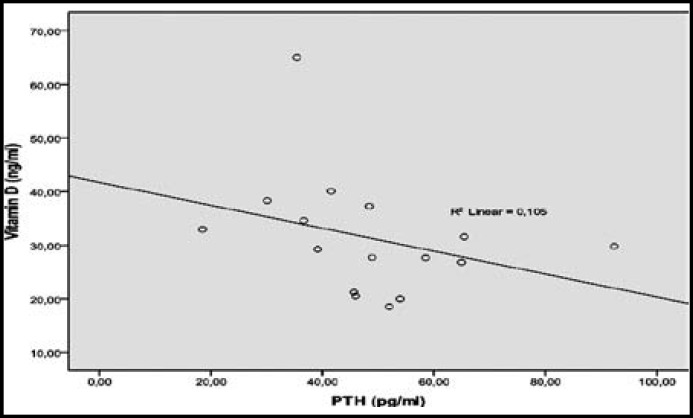
Negative correlation between postreplacement vitamin D (ng/ml) and PTH (pg/ml) levels

The baseline mean vitamin D levels were 15.1±6.6 ng/ml and Vitamin D deficiency (<25ng/ml) was found in 93.9% of the patients. Post replacement mean Vitamin D levels were 29.4±10.8 (Table-II). Baseline and postreplacement Vitamin D levels were not significantly associated with age or gender.

When the pre and post-treatment metabolic parameters were compared, serum vitamin D levels increase was statistically significant (p<0.001) and fasting insulin, HbA1c, HOMA-IR values decreased statistically significant after the treatment (n=66) (Table-II). Distribution of baseline and post-replacement Vitamin D levels were shown in Fig.1. A significant negative correlation was found between postreplacement vitamin D levels and PTH as expected (Fig.2).

## DISCUSSION

The prevalence of Vitamin D deficiency increases gradually, and new guidelines to increase vitamin D to optimal levels are prepared.^18^ Serum 25(OH)D levels are determined by environmental factors, particularly ultraviolet, and by, while having lesser importance, the intake of Vitamin D from food.^6^ Among the most important factors of Vitamin D deficiency, low dairy product consumption due to widespread lactose intolerance in our country, and living indoor places, and wearing clothes covering majority of the body, particularly in women, may be listed.^19^ Also diseases arising due to gastrointestinal absorption disorders such as coeliac disease and iron and vitamin B12 deficiency-related anemia, may be the cause of Vitamin D deficiency epidemic. Therefore, it is recommended particularly for those working indoors to benefit from the sunlight and exercise and to increase the consumption of dairy products as well as fish.^18^

The relation of Vitamin D deficiency and glucose metabolism, insulin resistance and metabolic syndrome, is inconsistent in the literature. In a study published in 2011, it was stated that low vitamin D levels increased prediabetes risk, and in healthy adolescent males, the insulin levels decreased as the vitamin D level increased.^8^ Pittas et al, reported that the type 2 diabetes risk was lower for those whose 25(OH)D levels were higher.^9^ On the other hand, in a study carried out in postmenopausal women, it has been reported that low vitamin D levels were not associated with diabetes risk.^20^

The results of studies evaluating the effects of Vitamin D replacement are also inconsistent. The Women’s Health Initiative Study results did not show the positive effect of daily supplementation of 1000 mg of calcium and 400 IU of Vitamin D for a period of 7 years in preventing the risk of type 2 diabetes.^21^ Similarly in a study made in our country, no relation was found between insulin resistance and Vitamin D deficiency.^22^ On the other hand, Von Hurst et al, reported that there was a decrease in insulin resistance following high dose Vitamin D for a period of 6 months in Vitamin D deficient women.^23^ We also found that serum insulin, HbA1c and HOMA IR levels have decreased significantly by the replacement in our study. 'Although we have seen some reduction in body weight in the patient group at the end of the study, the difference from baseline was not significant and their BMI values did not significantly changed, (most likely due to poor compliance of the patients). This result suggests that the improvement in glucose parameters resulted from Vitamin D replacement, independently of changes in BMI and/or body weight.

In studies researching the extraskeletal impacts of vitamin D supplementation, generally high doses such as 2000-7000 IU per day were used, and it is also recommended to reach the optimal levels above 40 and even 50 ng/dl by some authors^16^^,^^22^ Besides, the possibility of individual/racial differences such as receptor polymorphism affecting the Vitamin D metabolism and its deficiency status should be taken into consideration.

One of the limitations of our study was that the compliance of patients to the recommended lifestyle modifications and treatment protocol were fully depended on patients’ feedbacks. Secondly, in the beginning of the study, we planned to design the study with 2 groups, one with Vitamin D supplementation, the other without supplementation but most of the patients were found to be vitamin D-deficient, and the number of patients whose vitamin D levels were sufficient was too small to make the comparisons. And lastly, Vitamin D replacement doses may have been lower than the latest guideline recommendations.^18^

In conclusion, Vitamin D deficiency was very common in our patient population, consistent with recent studies in our country.^24^^-^^26^ However, improvements in glucose parameters such as HbA1c, HOMA IR and insulin levels were observed following the Vitamin D replacement. Our study also showed that Vitamin D deficiency is an important public health problem in our country, just as the case throughout the world. Accordingly, supplementing the food with Vitamin D and raising awareness for the benefits of sunlight should be considered since this may help reduce the risk of glucose metabolism disorders.

Determining the optimal Vitamin D requirements has become an important need for our country. It is very important to eliminate Vitamin D deficiency and maintain optimal levels by the use of easily accessible, inexpensive and safe Vitamin D preparations. Screening for vitamin D deficiency, particularly in high risk groups might be a cost-effective and important step for avoiding the risk of diabetes and other chronic diseases besides protecting bone health.^18^ Screening for Vitamin D deficiency, particularly in high risk groups might be a cost-effective and important step for avoiding the risk of diabetes and other chronic diseases besides protecting bone health.^22^ Therefore, we believe, further long-term placebo controlled prospective studies are needed in order to (1) find out the reasons which may explain the frequency of deficiency, such as adipose tissue, individual absorption differences and (2) reveal genetic and/or environmental factors and drugs that might affect serum 25(OH)D concentrations.
